# What Are the Barriers for Uptake of Antiretroviral Therapy in HIV-Infected Tuberculosis Patients? A Mixed-Methods Study from Ayeyawady Region, Myanmar

**DOI:** 10.3390/tropicalmed5010041

**Published:** 2020-03-09

**Authors:** Naychi Htet Htet Lin Aung, Kyaw Thu Soe, Ajay M.V. Kumar, Saw Saw, Si Thu Aung

**Affiliations:** 1 Regional Public Health Department, Pathein 10012, Ayeyawady Region, Myanmar; 2 Department of Medical Research (Pyin Oo Lwin Branch), Ministry of Health and Sports, Pyin Oo Lwin 05081, Myanmar; kyawthusoe.dmr@gmail.com; 3 International Union Against Tuberculosis and Lung Disease (The Union), Paris 75006, France; akumar@theunion.org; 4 The Union South-East Asia Office, New Delhi 110016, India; 5 Yenepoya Medical College, Yenepoya, Mangaluru 575022, India; 6 Department of Medical Research (Head Quarter), Ministry of Health and Sports, Yangon 11191, Myanmar; sawsawsu@gmail.com; 7 Disease Control Division, Department of Public Health, Ministry of Health and Sports, Nay Pyi Taw 15011, Myanmar; sithuaung@mohs.gov.mm

**Keywords:** reasons for non-initiation, TB/HIV collaboration, sort it, implementation research, delays

## Abstract

Antiretroviral therapy (ART) coverage among HIV-infected tuberculosis (HIV-TB) patients has been suboptimal in Myanmar and the reasons are unknown. We aimed to assess the ART uptake among HIV-TB patients in public health facilities of Ayeyawady Region from July 2017–June 2018 and explore the barriers for non-initiation of ART. We conducted an explanatory mixed-methods study with a quantitative component (cohort analysis of secondary programme data) followed by a descriptive qualitative component (thematic analysis of in-depth interviews of 22 providers and five patients). Among 12,447 TB patients, 11,057 (89%) were HIV-tested and 627 (5.7%) were HIV-positive. Of 627 HIV-TB patients, 446 (71%) received ART during TB treatment (86 started on ART prior to TB treatment and rest started after TB treatment). Among the 181 patients not started on ART, 60 (33%) died and 41 (23%) were lost-to-follow-up. Patient-related barriers included geographic and economic constraints, poor awareness, denial of HIV status, and fear of adverse drug effects. The health system barriers included limited human resource, provision of ART on ‘fixed’ days only, weaknesses in counselling, referral and feedback mechanism, and clinicians’ reluctance to start ART early due to concerns about immune reconstitution inflammatory syndrome. We urge the national TB and HIV programs to take immediate actions to improve the ART uptake.

## 1. Introduction

Tuberculosis (TB) remains a major global problem, despite impressive progress over the past two decades. *Mycobacterium tuberculosis* causes TB and it is a leading cause of death among infectious diseases, ranking above HIV/AIDS. An estimated 10 million people fell ill with TB in 2018, with 8.6% among people living with HIV (PLHIV) [1]. An estimated 1.2 million people died due to TB in the same year and additional 251,000 deaths among HIV-infected TB patients [1]. While Africa remains the epicenter of HIV-associated TB epidemic, the South-East Asian region accounts for 16% of the global burden [1]. Myanmar, a lower-middle income country that is situated in South-East Asia region, is one of the 30 countries with a high burden of HIV-associated TB [1,2,3]. 

To address the dual epidemic of TB and HIV, the World Health Organization (WHO) recommends a set of collaborative activities [4]. These include HIV testing of all patients with presumptive and active TB, cotrimoxazole preventive therapy (CPT), and antiretroviral therapy (ART) to all HIV-infected TB patients, irrespective of CD4 count. Implementing these measures is crucial to end the TB and HIV epidemics by 2030, as enunciated in the sustainable development goals of the United Nations [5].

Myanmar implements all of the WHO-recommended TB/HIV collaborative activities nationally and there has been a steady progress over the past ten years [6]. HIV testing among TB patients has progressively increased and it stood at 90% for the year 2017. While more than 80% of all HIV-infected TB patients received CPT, only 63% received ART, far below the global and national targets [7]. The ART uptake is even lower, at 36% in Ayeyawady Region, one of the highest TB-HIV burden areas in Myanmar [8]. This is deeply concerning, as non-initiation of ART is associated with higher death rates [9,10]. This is reflected in higher case fatality among HIV-positive TB patients (~20%) in Ayeyawady Region when compared to those who are not HIV-positive (~5%) [11].

Several measures have been taken since early 2017 to decentralize the ART services to township level health facilities in the country to provide ART for HIV-infected TB patients. There is a need to assess if these measures have led to an improvement in ART uptake. Further, it is not clear as to why all HIV-infected TB patients are not receiving ART, despite the policy that all should be started on ART, irrespective of CD4 count. There has not been any published evidence on this issue from Myanmar [12]. Understanding the reasons for non-initiation of ART has been identified as one of the priority research questions by the national TB program [13].

Hence, we undertook a mixed-methods operational research to i) assess the ART uptake among HIV-infected TB patients registered in the public health facilities of Ayeyawady Region of Myanmar between July 2017 and June 2018, ii) assess the demographic and clinical factors that are associated with non-initiation of ART, and iii) explore the barriers for non-initiation of ART from patients’ and providers’ perspective.

## 2. Materials and Methods 

### 2.1. Study Design

This was an explanatory mixed-methods study with a quantitative component (cohort analysis of secondary data routinely collected by programs), followed by a descriptive qualitative component (thematic analysis of interviews) [14].

### 2.2. Study Setting

*General Setting*

Myanmar is situated in South-East Asia and it is administratively divided into 15 States and Regions, 74 districts, and 330 townships. The population is 54 million and about 70% live in rural areas [15]. The study was conducted in Ayeyawady Region (population of 6.2 million), a coastal region of Myanmar [16]. The geographical terrain is difficult with limited transportation facilities. Only boats connect many areas. Most people live in rural areas (86%).

*Specific Setting*

There are six districts and 26 townships in Ayeyawady Region. There are a total of 442 public health facilities: 120 general hospitals, 26 township TB centres, 241 rural health centres, four urban health centres, 42 maternal and child health centres, and nine school health centres.

*National TB Programme (NTP)*

Every township has a township TB centre that offers laboratory tests (sputum smear microscopy, Xpert MTB/RIF) and chest radiography for patients presenting with presumptive TB. Presumptive TB among PLHIV are preferentially offered Xpert MTB/RIF assay, which is only available at the district level. Those diagnosed with TB are started on anti-TB treatment at township TB centres while using standard regimens that are in line with the WHO TB treatment guidelines [17].

*National AIDS Programme (NAP)*

All of the patients with presumptive and active TB are offered HIV testing using rapid diagnostic tests. Initially, NAP staff did HIV testing, but, since 2014, NTP staffs complete HIV screening and trained laboratory technicians working for NAP and the general health system undertake confirmation. All of the laboratories function under a system of external quality assurance. HIV-infected TB patients are referred to the ART centres to receive CPT and ART, irrespective of CD4 count. While HIV testing services are available at all health facilities, ART services are available at 24 (out of 26) townships. CD4 testing facility is only available in five townships. TB treatment is initiated first, followed by ART as soon as possible within 2–8 weeks of TB treatment. Patients with profound immunosuppression (such as CD4 count < 50 cells/µL) are expected to receive ART immediately within the first two weeks of initiating TB treatment [17].

NTP and NAP both operate predominantly as vertical programs and are not yet fully integrated within the general health system. This means that patients do not get all of the services under one roof. They have to travel from one place to another, often on different days to access TB and HIV services. All of the services are provided free of charge to the patients.

*Recording and Reporting*

For monitoring of TB patients, paper-based treatment cards and registers using standardized definitions for case finding and treatment outcomes, which are in accordance with the WHO guidelines used. HIV status is recorded in the laboratory register, tuberculosis treatment card, and Township TB register, with the latter also recording whether CPT and ART has been initiated. 

### 2.3. Study Population, Sampling and Sample Size

*Quantitative*

All of the HIV-infected TB patients registered in the township TB centres and Pathein general hospital from Ayeyawady Region and receiving first-line anti-TB treatment from July 2017 to June 2018 were included. Transfer-in patients were excluded to avoid the double counting of patients.

*Qualitative*

The study population included a sample of health care providers and HIV-infected TB patients. Purposive sampling was employed and saturation was used to guide sample size. For the sampling of providers, we first selected townships based on three characteristics: the TB-HIV burden, ART coverage, and availability of ART services. Subsequently, from each township we selected one TB coordinator and one HIV focal person for Key Informant Interview (KII). In addition, one specialist physician was also interviewed, wherever available. Thus, a total of 22 KIIs were conducted. None of the providers contacted refused to be interviewed.

For patient interviews, we first abstracted the list of HIV-infected TB patients who did not receive ART from the registers. Among them, we excluded those who were reported died or lost to follow-up (LFU) and those aged < 18 years. The lists of remaining patients were given to focal persons of TB and HIV teams from respective health facilities. We found that many patients had no address or had provided wrong address and phone numbers when attempts were made to contact. Some patients had moved to other places and could not be tracked, while some patients refused interviews. Therefore, we could only interview a total of five patients—three who did not receive ART and two who received ART. 

### 2.4. Data Variables, Data Sources and Data Collection Procedure

*Quantitative*

Data with respect to demographic (age, sex, referred person) and clinical characteristics (type of TB, site of TB, ART status, dates of initiation of TB treatment, and ART initiation) of HIV-infected TB patients were first abstracted from the existing electronic township TB registers at the Regional Office. We also reviewed the TB treatment cards, ART treatment cards, pre-ART registers, and ART registers to i) collect additional variables (CD4 counts), ii) complete the data missing in electronic TB registers, and iii) validate and update the ART status, with the help of TB and HIV focal points in the townships. We extracted aggregate numbers of TB patients registered during the study period and the numbers whose HIV status was ascertained from the program TB report.

*Qualitative*

The principal investigator (NC, female medical doctor, with an MBBS and MPTM degrees and working as assistant director for the national TB and leprosy programs in Ayeyawady Region) and co-investigator (KTS, male medical doctor with an MBBS and MPHM degrees working as a research scientist in the Department of Medical Research) conducted the interviews. Both the interviewers are well trained and experienced in qualitative research methods and have good knowledge of the local context and programs. While both conducted the patient interviews, all of the provider interviews were conducted by KTS. Since KTS is not a part of the hierarchy within the national TB or HIV program, this might have helped the participants to express freely. Interviews were done face-to-face (except one provider who was interviewed over telephone) at a time and place that is convenient to the participants while using interview guides, which were pilot-tested and amended before use. Interview guides were updated based on the initial analysis of quantitative and qualitative results. Interviews were conducted in the local Burmese language and audio recorded after receiving consent from the participants. Verbatim notes were also taken during the interview. All of the interviews were conducted in the health facility and only the interviewer and interviewee were present during the process. Each interview lasted for approximately 30–60 minutes. After the interview, a summary was read back to the participants as a way of member checking.

Data were collected between November 2018 and May 2019.

### 2.5. Data Analysis

*Quantitative*

Data analysis was conducted while using EpiData (v2.2.2.186, EpiData Association, Odense, Denmark) and Stata software (v12.1, Texas, USA). The key outcome variable was ‘non-initiation of ART’, defined as having not received ART anytime during TB treatment. For this analysis, only ART-naïve patients at the start of TB treatment were included. We excluded patients receiving ART prior to TB treatment. Adjusted relative risks along with 95% confidence intervals were calculated in a multivariable log-binomial or modified Poisson regression model with robust variance estimates (in case convergence was not obtained with log-binomial models) to adjust for confounding and identifying the independent factors associated.

*Qualitative*

We transcribed all of the interviews in Burmese language the same day. Thematic analysis by manual coding was then carried out by NC and KTS independently to generate various categories or themes under the broad topics: patient-related and health system-related barriers [18,19]. We used a combination of deductive and inductive coding. A third investigator (AMVK) further reviewed the analysis to reduce the subjectivity in analysis and increase the interpretive credibility. Any difference between the researchers was resolved by discussion and consensus.

### 2.6. Ethics and Consent 

We obtained ethics approval from the Ethics Review Committee, Department of Medical Research, Ministry of Health and Sports, Myanmar (Ethics/DMR/2019/022) and the Ethics Advisory Group of International Union Against Tuberculosis and Lung Disease, Paris, France (47/18). Permission to conduct the study was obtained from the National Tuberculosis Program and National AIDS Program, Ministry of Health and Sports, Myanmar. Written informed consent was obtained from all of the interviewed participants.

## 3. Results

### 3.1. Quantitative

There were a total of 12,447 TB patients registered. Of them, 11,057 (89%) were tested for HIV and 627 (5.7%) were HIV-positive. Table 1 describes the characteristics of HIV-infected TB patients. There were 415(66%) males and the mean (SD) age was 36 (13) years. Most of them were new TB cases (91%) and had pulmonary TB (95%). Among the 33 (5%) extra pulmonary TB patients, 32 had lymph node tuberculosis, and one had TB meningitis. CD4 count data were not recorded in 405 (65%) of the patients. Of those with CD4 count data, the median (IQR) CD4 count was 110 (53–226) cells/µL.

*ART Uptake*

**Figure 1:**
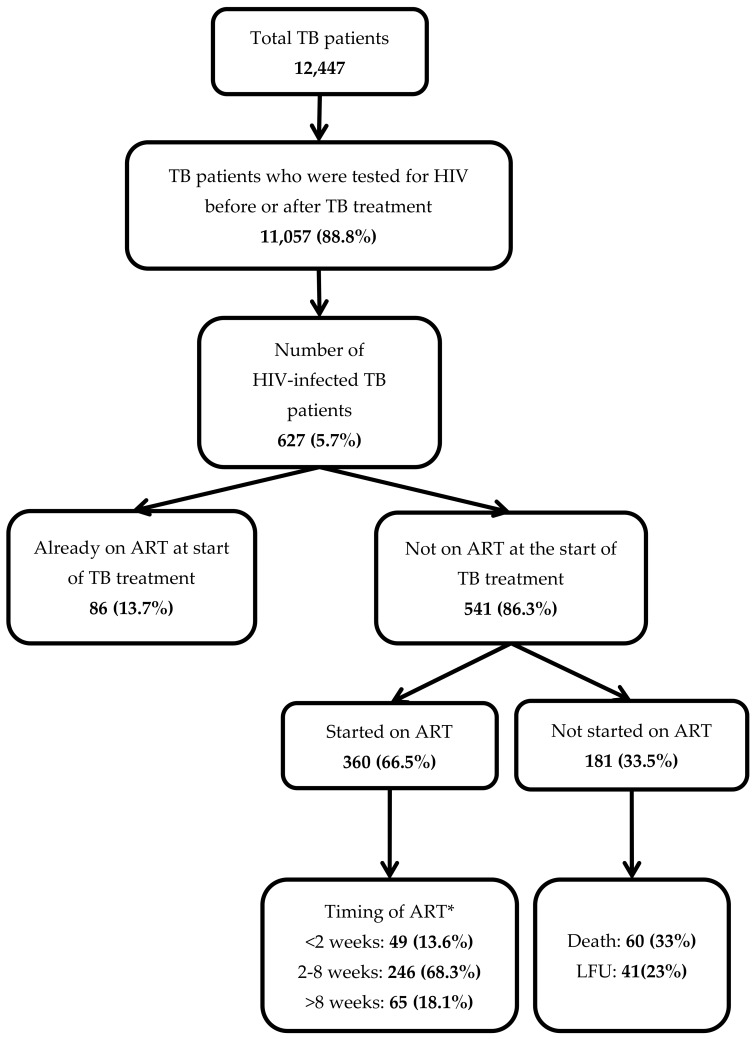
Antiretroviral therapy (ART) uptake and its timing in a cohort of HIV-infected tuberculosis (TB) patients registered in the public health facilities of Ayeyawady Region of Myanmar, between July 2017 and June 2018.

Of the 627 HIV-infected TB patients, 86 (14%) were already on ART prior to TB treatment. Of the remaining 541 patients who were not on ART at the start of TB treatment, 360 (67%) were initiated on ART during TB treatment (Figure 1). Thus, a total of 446 (71%) received ART during TB treatment. Among the 181 patients who did not receive ART, 60 (33%) died and 41 (23%) were lost to follow-up.

*Timing of ART initiation*

The median duration between start of TB treatment and ART initiation was 28 days (IQR: 18–45 days). Of those started on ART, 295 (82%) started within eight weeks of start of TB treatment as per the guidelines.

*Factors Associated with Non-initiation of ART*

In adjusted analysis, female patients, self-referred patients and those with missing CD4 counts had a higher risk of non-initiation of ART during TB treatment (Table 2).

### 3.2. Qualitative

**Figure 2:**
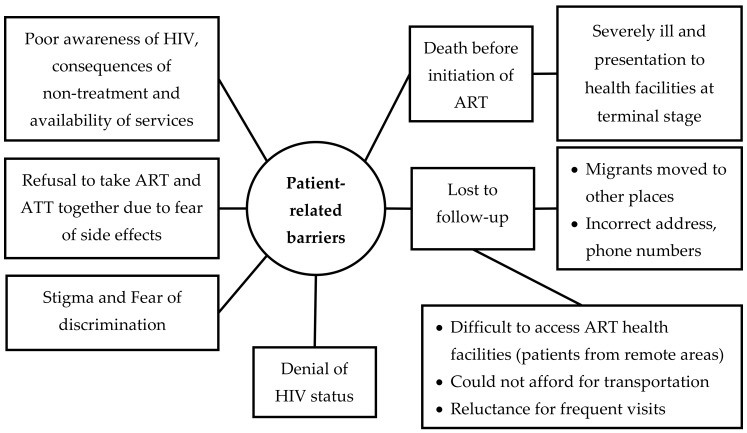
Non-hierarchical thematic map showing patient-related barriers in uptake of antiretroviral therapy among HIV-infected tuberculosis patients registered in the public health facilities of Ayeyawady Region of Myanmar, between July 2017 and June 2018.

**Figure 3:**
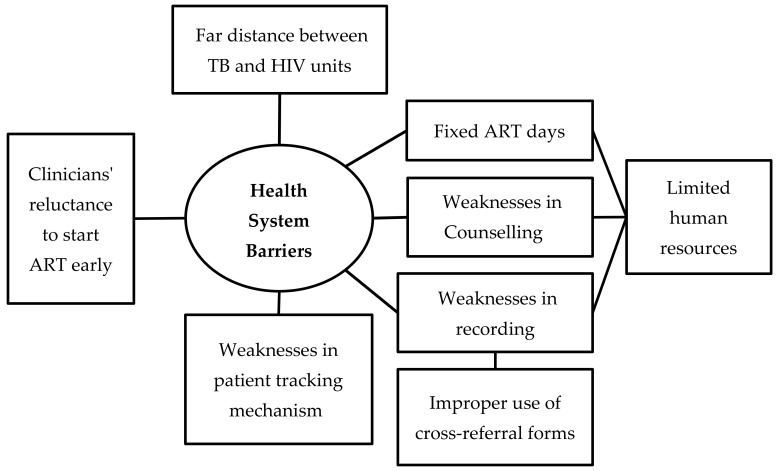
Non-hierarchical thematic map showing health system barriers in uptake of antiretroviral therapy among HIV-infected tuberculosis patients registered in the public health facilities of Ayeyawady Region of Myanmar, between July 2017 and June 2018.

Barriers were multi-factorial and inter-related with each other. We organized the barriers under two broad themes—patient-related barriers and health system-related barriers (Figure 2 and Figure 3). The barriers that are summarized here reflect the perspectives of both the patients and health care providers. The verbatim quotes (translated in English) are italicized and placed within double quotes.

*Patient-Related Barriers*

*Death before Initiation of ART*

The providers reported that many HIV-infected TB patients presented to the hospitals in the terminally ill stage and died before ART could be initiated.

*“Some patients came in severely ill stage... very ill... terminal stage... after anti-TB treatment, we could not even start ART, many cases expired”*

(Specialist).

*Lost to Follow-Up (LFU)*

The providers mentioned LFU as another major reason for non-initiation of ART. They reported that patients were lost at different points in the care pathway from HIV diagnosis to the completion of anti-TB treatment. Some patients were migrant labourers who had come to the city temporarily for work and moved back to their native places on completion of work. Such patients could not be traced, due to lack of accurate addresses and phone numbers. The providers opined that some of these patients might be receiving ART at other hospitals in other regions, but could not be confirmed.

*“Mainly the patients who were migrants... they came to work as salt workers, fisher men. They went back to their places before getting ART”*

(TB coordinator).

Other reasons reported for LFU included economic constraints faced by the patients and geographical constraints in Ayeyawady Region which required transport by boats. A patient reported that he could not afford the transportation costs required to visit the health facilities, especially when frequent visits were required for counselling before starting ART.

*“… they (HIV unit) asked me to come back... but...(sadly) I was unable to afford transportation charges in the third time… so... I could not go to them regularly… but I ate some hearsay leaves to cure my disease”*

(Patient).

It was reported that patients who lived in the hard-to-reach geographical areas with difficult and limited transportation facilities were more likely to be LFU.

*“It is the worst in the raining season, boats were prohibited for transportation, patients failed to come back to health facilities in time”*

(Specialist).

*“Because of the boats schedule (12:00 noon)… if patients were asked to visit ART clinics, they did not want to go as they had to catch the boats to be able to go back their homes... to stay overnight here, they did not have money”*

(TB coordinator).

*Stigma and Fear of Discrimination*

The providers felt that some patients did not want their family and anyone from their communities to know about their HIV disease. Hence, they did not visit the HIV unit from their towns to receive the care including ART.

*“We referred the patients to HIV unit next to our clinics for further ART management, but, patients were shy to sit and wait at HIV unit… and they went back without visiting”*

(TB team leader).

*Poor Awareness*

There was poor awareness in some patients about the nature of the disease, its consequences if untreated and the effectiveness of the life-saving nature of ART. Some patients resorted to unproven local remedies from unqualified providers.

*“Patients from remote areas with difficult transportation, they took treatment at their villages with quacks, they did not know TB or HIV, only when they were severely ill... they reached to hospitals”*

(Specialist).

A patient reported that though he was referred, he was not able to locate the correct place to access ART.

*“At the first time, I reached to the wrong health facility… as I have not visited this town before… It was very difficult for me to find the place”*

(Patient).

*Denial of HIV Status*

A patient reported that he did not trust the results of health facilities regarding his HIV positive status.

*“I would say straight. I did not believe in hospitals… that’s why I did not come back for next visit”*

(Patient).

*Fear of Side Effects*

The providers reported that some patients refused to take ART while they were on anti-TB drugs because of fear of side effects of both drugs.

*“Although we (doctors) counseled the patients the need of ART, patients refused… they would like to finish anti-TB first for six months, then would continue ART… as they were afraid”*

(TB team leader).

*Health System Barriers*

*Limited Human Resource and Suboptimal Collaboration*

TB and HIV units both have deficiencies in health staffs, including specialists, medical doctors, nurses, laboratory technicians, and counselors. This meant that the existing staff were burdened with multiple responsibilities that led to suboptimal co-ordination between TB and HIV services.

*“I did not know who was the focal person from HIV unit, I heard that THN (Township health nurse) was the focal person, but there were frequent turnover of nurses... I did not know who were responsible for recording and reporting for HIV patients, so difficult to receive feedback”*

(TB coordinator).

*Fixed ART Days*

ART services were provided on fixed days once a week or once in two weeks because of limited number of specialists and medical doctors. This contributed to LFU.

*“They (HIV unit) received patients for ART at the second WEDNESDAY and last WEDNESDAY, two times a month. When we referred our patients for ART, and it was not ART provision day, patients were given the scheduled appointment, but, patients couldn’t come back because of their difficulties”*

(TB coordinator).

*Weaknesses in Reporting and Recording and Patient-Tracking Mechanisms*

The providers mentioned that the cross-referral forms were not always used to document referral from TB unit to the HIV unit. This, they opined might have led to under-recording and under-reporting of ART status of HIV-infected TB patients.

*“They (TB and HIV focal persons) met only once in three months to track the HIV and ART status of TB HIV”*

(District Medical Officer).

*“As (TB and HIV units) were close, we did not use cross-referral forms when transferring TB-HIV patients. We just made verbal communication”*

(TB coordinator).

*Weaknesses in Patient Counseling*

The providers felt that counseling to patients needed to be strengthened, so that they fully understand the importance of the disease and the consequences of not getting treated with ART. In some cases, patients thought they had to take anti-TB drugs only.

*Clinicians’ Reluctance to Start ART Early*

Clinicians that were involved in this study reported that they were reluctant to initiate ART without a CD4 count and other baseline investigations, as they thought it was necessary to monitor progress to treatment. Some of the physicians were concerned about the possibility of development of immune reconstitution inflammatory syndrome (IRIS), when both TB treatment and ART were simultaneously started. Physicians delayed the initiation of ART in patients who were severely ill, malnourished, and with several co-morbidities, because they felt that such patients might not be able to tolerate the pill burden and consequent adverse drug reactions.

*“Some patients would like to take ART, but CD4 count was below 50, so, to avoid IRIS we waited for some time to initiate ART”*

(Specialist).

## 4. Discussion

This is the first study from Myanmar to assess the barriers to uptake of ART among HIV-infected TB patients and fills an important knowledge gap about TB/HIV collaboration in the country. We discuss the key findings below.

We found that about seven in ten HIV-infected TB patients received ART during TB treatment. When compared to previous reports from the same region, where in only about 45% received ART, this is a marked improvement. This might be partly because of the decentralization of ART services to township level and partly because of improved data triangulation processes being employed as part of the research study. Nevertheless, this is still far away from the global (100%) and national (85%) targets [7] and the experiences in other countries from African region (~90%) and Asia (India 90%, Vietnam 93%, Bangladesh 94%) [1]. It is possible that some of the patients (especially migrant labourers) may have returned to their native places and they may be receiving ART in other regions. This might have underestimated the ART uptake in our study and is a limitation.

There were several barriers that were identified for non-initiation of ART. These are similar to those identified elsewhere [20]. Death and LFU were the key barriers and accounted for more than half of the patients not initiated on ART—this was observed both in quantitative and qualitative data. Some of the LFU cases may be unreported deaths. 

Many patients were severely ill by the time that they reached health facilities. Approximately 25% of the patients had a CD4 count below 50/µL. These indicate gross delays in the diagnosis of HIV and in the diagnosis of TB among PLHIV. Urgent measures need to be taken to prevent these delays—which include improving the HIV test uptake, which has been reported to be suboptimal in key populations and presumptive TB patients, implementation of ‘test and treat’ strategy, which has great potential for the prevention of HIV-associated TB, [21,22] and early diagnosis of TB while using Xpert MTB/RIF assay [23]. While all of these policies are in place in Myanmar, the extent of implementation varies. 

Xpert MTB/RIF assay is recommended as the first test of choice to diagnose TB among PLHIV. There are challenges for accessing this assay, because Gene Xpert machines are only available at the district level, although sputum collection and transport mechanisms are in place at all townships. This technology needs to be scaled up further. There are newer versions of Xpert, such as Xpert Ultra (which is more sensitive than Xpert) and Xpert Omni, which is portable and helps in decentralizing this technology at the primary health care level [1,24]. NTP needs to consider deploying these tools for PLHIV to enable early diagnosis of TB. 

Another barrier to ART uptake was LFU—which was, in turn, related to geographical constraints, economic constraints, and health-system related barriers. Ayeyawady Region is a delta region with many areas that are only accessible by boat transport. Many a time, the boat timings are not convenient for patients to attend the health facilities. Decentralizing the ART services and integrating them within the general health system, so that ART is available at all the health facilities, may be a way forward to reduce long travel by the patients. Qualitative interviews indicated that many patients were poor and could not afford travel costs to the ART centre. NAP must make efforts to reimburse travel costs, at-least for those patients who are living below the poverty line. Such measures are already in place in Myanmar for multidrug-resistant TB patients and they can be easily expanded to HIV-TB patients. 

An important health-system related barrier related to LFU was the system of providing ART services on ‘fixed’ days only (once a week or once in two weeks), due to human resource constraints. This meant that the patients had to travel multiple times and on different days to access TB and HIV services. If patients missed the fixed days, they had to wait for the next scheduled day. Quite often, HIV services and TB services were not located at the same place, requiring the referral of patients from one place to another. All these challenges might have facilitated LFU and delays in ART initiation. These findings call for the full integration of TB and HIV services at all the health facilities, which has been shown to be effective in improving ART uptake [25,26,27].

One of the key barriers was the reluctance on the part of the physicians to start early ART due to concerns about development of IRIS, especially in severely ill PLHIV with low CD4 counts. This is a genuine concern, as many studies have shown that the incidence of IRIS is higher in HIV-infected TB patients started on early ART as compared to late ART [28,29,30]. However, IRIS events are rarely fatal and many studies, including three clinical trials, namely SAPiT, STRIDE, and CAMELIA, have unequivocally demonstrated the survival benefits of early ART in HIV-infected TB patients, especially in those with CD4 counts <50/µL [29,30,31,32,33]. There was no survival benefit of early ART in patients with HIV-associated TB meningitis, but even in this study, there was no additional harm due to early ART [34]. Thus, the benefits of early ART far outweigh the risks and it is not appropriate to delay ART in an effort to prevent IRIS. It is imperative that the physicians be vigilant, so that IRIS is detected early and treated appropriately. 

Is there a way to prevent IRIS? A recent trial showed that providing prophylactic prednisone for four weeks along with ART resulted in a lower incidence of tuberculosis-associated IRIS compared to placebo, without evidence of an increased risk of severe infections or cancers [35]. The NTP and NAP in Myanmar should consider using this strategy. This might increase the confidence of the physicians to manage the severely ill patients.

*Strengths*

Our study was conducted under program conditions, including all eligible patients (without any sampling) and it covered one full region of Myanmar. Thus, we feel that the results accurately reflect the programmatic realities. We used a mixed methods design, which helped in a comprehensive assessment of the issue. We adhered to ‘Strengthening the Reporting of Observational Studies in Epidemiology’ (STROBE) and consolidated the guidelines for reporting qualitative research (COREQ) for reporting [36,37].

*Limitations*

As the study was conducted only in one region, the findings have to be generalized beyond the region with caution. While the health-system related barriers might be similar, some of the patient-related barriers (like geographical constraints) may differ. As described earlier, we had challenges in recruiting patients for interviews and as a result we might not have achieved saturation of the findings from their perspective. Data with respect to CD4 counts were missing in two-thirds of patients. This needs to be rectified.

## 5. Conclusions

In this mixed-methods operational research, we found that about seven in ten HIV-infected TB patients registered in the public health facilities of Ayeyawady Region of Myanmar in 2017–18 received ART during TB treatment. This is a marked improvement when compared to previous years. More than half of all patients who did not receive ART either died or were lost of follow-up. Several patient-related and health-system related barriers were identified. Addressing these barriers is crucial in improving ART uptake among HIV-infected TB patients and to end the TB and HIV epidemics.

## Figures and Tables

* duration between start of TB treatment and start of ART; TB: Tuberculosis; HIV: Human Immunodeficiency Virus; ART: Antiretroviral Therapy; LFU: Loss to follow-up.

**TB:** Tuberculosis; **HIV:** Human Immunodeficiency Virus; **ART:** Antiretroviral Therapy; **LFU:** Loss to follow-up; **ATT**: Anti-tuberculosis treatment

**Table 1:** Demographic and clinical characteristics of HIV-infected TB patients registered in the public health facilities of Ayeyawady Region of Myanmar, between July 2017 and June 2018.

Variable	N	(%)
**Total**	**627**	**(100)**
**Age**		
0–14 years	53	(8.5)
15–34 years	218	(34.8)
35–54 years	302	(48.2)
≥55 years	54	(8.6)
**Sex**		
Male	415	(66.2)
Female	212	(33.8)
Referred Person *		
Health Staff	526	(83.9)
Private Practitioner	46	(7.3)
Self-referral	53	(8.5)
Other	2	(0.3)
Type of TB		
New	573	(91.4)
Previously treated	54	(8.6)
**Site of TB**		
Pulmonary	594	(94.7)
Extra pulmonary	33	(5.3)
**CD4 Count (cells/µL)**		
<50	54	(8.6)
50–199	100	(15.9)
200–349	38	(6.1)
350–499	16	(2.6)
≥500	14	(2.2)
Missing	405	(64.6)

TB: Tuberculosis; HIV: Human Immunodeficiency Virus; ART: Antiretroviral Therapy; * referred for TB diagnosis.

**Table 2:** Factors that are associated with non-initiation of ART among ART-naïve HIV-infected TB patients registered in the public health facilities of Ayeyawady Region of Myanmar, between July 2017 and June 2018 (N = 541).

Variables	Total	Non-Initiation of ART		uRR (95% CI)	aRR (95% CI)
		N	(%)		
**Total**	541	181	(33.5)		
**Age**					
0–14 years	46	19	(41.3)	1.35 (0.92–1.99)	1.07 (0.86–1.33)
15–34 years	182	63	(34.6)	1.13 (0.86–1.48)	1.09 (0.92–1.28)
35–54 years	265	81	(30.6)	Ref	Ref
≥55 years	48	18	(37.5)	1.23 (0.82–1.85)	1.08 (0.91–1.27)
**Sex**					
Male	365	110	(30.1)	Ref	Ref
Female	176	71	(40.3)	**1.34 (1.06–1.70)**	**1.36 (1.13–1.64)**
Referred Person *					
Health Staff	456	147	(32.2)	Ref	Ref
Private Practitioner	33	9	(27.3)	0.85 (0.48–1.50)	1.01 (0.58–1.78)
Self-referral	50	24	(48.0)	**1.49 (1.08–2.05)**	**2.00 (1.68–2.39)**
Other	2	1	(50.0)	1.55 (0.39–6.24)	1.35 (0.22–8.19)
**Type of TB**					
New	503	168	(33.4)	Ref	Ref
Previously treated	38	13	(34.2)	1.02 (0.65–1.62)	**0.99 (0.99–0.99)**
**Site of TB**					
Pulmonary	514	171	(33.3)	Ref	Ref
Extra pulmonary	27	10	(37.0)	1.11 (0.67–1.85)	1.14 (0.74–1.76)
**CD4 count (cells/µL)**					
<200	134	24	(17.9)	Ref	Ref
≥200	59	10	(16.9)	0.95 (0.48–1.85)	0.89 (0.45–1.77)
Missing	348	147	(42.2)	**2.36 (1.61–3.46)**	**2.54 (1.73–3.72)**

TB: Tuberculosis; HIV: Human Immunodeficiency Virus; ART: Antiretroviral Therapy; uRR: Unadjusted risk ratio; aRR: adjusted risk ratio; CI: confidence intervals; RRs in bold are statistically significant (*p* value < 0.05); * referred for TB diagnosis.

## References

[1] World Health Organization (2019). Global TB Report 2019.

[2] Aung Z.Z., Saw Y.M., Saw T.N., Oo N., Aye H.N.N., Aung S., Oo H.N., Cho S.M., Khaing M., Kariya T. (2018). Survival rate and mortality risk factors among TB-HIV co-infected patients at a HIV-specialist hospital in Myanmar: A 12-year retrospective follow-up study. Int. J. Infect. Dis..

[3] World Health Organization WHO Meeting Report of a Technical Expert Consultation: Non-inferiority analysis of Xpert MTB/RIF Ultra compared to Xpert MTB/RIF. http://www.who.int/tb/publications/2017/XpertUltra/en/.

[4] World Health Organization (2012). World Health Organization WHO Policy on Collaborative TB/HIV Activities. Guidelines for National Programmes and Other Stakeholders.

[5] United Nations Transforming our World: Sustainable Development Goals. https://sustainabledevelopmen.orgt.un.org/sdg3.

[6] Theingi P., Harries A.D., Wai K.T., Shewade H.D., Saw S., Win T., Thein S., Kyi M.S., Oo H.N., Aung S.T. (2017). National scale-up of tuberculosis-human immunodeficiency virus collaborative activities in Myanmar from 2005 to 2016 and tuberculosis treatment outcomes for patients with human immunodeficiency virus-positive tuberculosis in the Mandalay Region in 2015. Trans. R. Soc. Trop. Med. Hyg..

[7] National Tuberculosis Programme, Ministry of Health and Sports, Government of Myanmar (2016). National Strategic Plan for TB (2016-2020).

[8] National Tuberculosis Program (Myanmar) (2018). Annual TB report 2016.

[9] Murray J., Sonnenberg P., Shearer S.C., Godfrey-Faussett P. (1999). Human immunodeficiency virus and the outcome of treatment for new and recurrent pulmonary tuberculosis in African patients. Am. J. Respir. Crit. Care Med..

[10] Churchyard G.J., Kleinschmidt I., Corbett E.L., Murray J., Smit J., De Cock K.M. (2000). Factors associated with an increased case-fatality rate in HIV-infected and non-infected South African gold miners with pulmonary tuberculosis. Int. J. Tuberc. Lung Dis.: Off. J. Int. Union Against Tuberc. Lung Dis..

[11] National Tuberculosis Programme, Ministry of Health and Sports, Government of Myanmar (2017). Annual TB Report of Ayeyawady Region, Myanmar.

[12] Kyi M.S., Aung S.T., McNeil E., Chongsuvivatwong V. (2018). Evolution of Tuberculosis/Human Immunodeficiency Virus Services among Different Integrated Models in Myanmar: A Health Services Review. Trop. Med. Infect. Dis..

[13] National Tuberculosis Program, Ministry of Health and Sports, Government of Myanmar (2017). Operational/Implementation Research Priorities.

[14] Creswell J., Plano Clark V. (2010). Designing and Conducting Mixed Methods Research.

[15] Department of Population, Ministry of Immigration and Population, Government of Myanmar (2015). The 2014 Myanmar Population and Housing Census. The Union Report: Census Report Volume 2.

[16] Department of Population, Ministry of Immigration and Population, Government of Myanmar (2015). The 2014 Myanmar Population and Housing Census: Ayeyawady Region.

[17] National Tuberculosis Programme, Ministry of Health and Sports (2017). Gove Guidelines for the Programmatic Management of TB/HIV in Myanmar.

[18] Attride-Stirling J. (1999). Thematic network: An analytic tool for qualitative research. Qualit. Res..

[19] Vaismoradi M., Turunen H., Bondas T. (2013). Content analysis and thematic analysis: Implications for conducting a qualitative descriptive study. Nurs. Health Sci..

[20] Wajanga B.M.K., Peck R.N., Kalluvya S., Fitzgerald D.W., Smart L.R., Downs J.A. (2014). Healthcare Worker Perceived Barriers to Early Initiation of Antiretroviral and Tuberculosis Therapy among Tanzanian Inpatients. PLoS ONE.

[21] Kaplan R., Hermans S., Caldwell J., Jennings K., Bekker L., Wood R. (2018). HIV and TB co-infection in the ART era: CD4 count distributions and TB case fatality in Cape Town. BMC Infect. Dis..

[22] Nagu T.J., Aboud S., Mwiru R., Matee M.I., Rao M., Fawzi W.W., Zumla A., Maeurer M.J., Mugusi F. (2017). Tuberculosis associated mortality in a prospective cohort in Sub Saharan Africa: Association with HIV and antiretroviral therapy. Int. J. Infect. Dis..

[23] National AIDS Program, Department of Public Health, Ministry of Health and Sports (2017). Guidelines for the Clinical Management of HIV infection in Myanmar.

[24] MSF Access Campaign Xpert OMNI FactSheet What to consider before Xpert Omni Implementation. https://www.ghdonline.org/uploads/OMNI_FACTSHEET_26012018_FINAL_qyHzL8O.pdf.

[25] Pathmanathan I., Pasipamire M., Pals S., Dokubo E.K., Preko P., Ao T., Mazibuko S., Ongole J., Dhlamini T., Haumba S. (2018). High uptake of antiretroviral therapy among HIV-positive TB patients receiving co-located services in Swaziland. PLoS ONE.

[26] Kerschberger B., Hilderbrand K., Boulle A.M., Coetzee D., Goemaere E., de Azevedo V., van Cutsem G. (2012). The Effect of Complete Integration of HIV and TB Services on Time to Initiation of Antiretroviral Therapy: A Before-After Study. PLoS ONE.

[27] Kyi M.S., Aung S.T., Oo H.N., Chongsuvivatwong V. (2019). Fully vs. partially integrated services for TB-HIV in Myanmar: A health services review and a cohort study. Int. J. Tuberc. Lung Dis.: Off. J. Int. Union Against Tuberc. Lung Dis..

[28] Naidoo K., Yende-Zuma N., Padayatchi N., Naidoo K., Jithoo N., Nair G., Bamber S., Gengiah S., El-Sadr W.M., Friedland G. (2012). The immune reconstitution inflammatory syndrome after antiretroviral therapy initiation in patients with tuberculosis: Findings from the SAPiT trial. An. Intern. Med..

[29] Abdool Karim S.S., Naidoo K., Grobler A., Padayatchi N., Baxter C., Gray A.L., Gengiah T., Gengiah S., Naidoo A., Jithoo N. (2011). Integration of antiretroviral therapy with tuberculosis treatment. N. Engl. J. Med..

[30] Havlir D. V, Kendall M.A., Ive P., Kumwenda J., Swindells S., Qasba S.S., Luetkemeyer A.F., Hogg E., Rooney J.F., Wu X. (2011). Timing of antiretroviral therapy for HIV-1 infection and tuberculosis. N. Engl. J. Med..

[31] Franke M.F., Robins J.M., Mugabo J., Kaigamba F., Cain L.E., Fleming J.G., Murray M.B. (2011). Effectiveness of early antiretroviral therapy initiation to improve survival among HIV-infected adults with tuberculosis: A retrospective cohort study. PLoS Med..

[32] Abdool Karim S.S., Naidoo K., Grobler A., Padayatchi N., Baxter C., Gray A., Gengiah T., Nair G., Bamber S., Singh A. (2010). Timing of initiation of antiretroviral drugs during tuberculosis therapy. N. Engl. J. Med..

[33] Blanc F.-X., Sok T., Laureillard D., Borand L., Rekacewicz C., Nerrienet E., Madec Y., Marcy O., Chan S., Prak N. (2011). Earlier versus later start of antiretroviral therapy in HIV-infected adults with tuberculosis. N. Engl. J. Med..

[34] Török M.E., Yen N.T.B., Chau T.T.H., Mai N.T.H., Phu N.H., Mai P.P., Dung N.T., Chau N.V.V., Bang N.D., Tien N.A. (2011). Timing of initiation of antiretroviral therapy in human immunodeficiency virus (HIV)—Associated tuberculous meningitis. Clin. Infect. Dis.: Off. Publ. Infect. Dis. Soc. Am..

[35] Meintjes G., Stek C., Blumenthal L., Thienemann F., Schutz C., Buyze J., Ravinetto R., van Loen H., Nair A., Jackson A. (2018). Prednisone for the Prevention of Paradoxical Tuberculosis-Associated IRIS. N. Engl. J. Med..

[36] von Elm E., Altman D.G., Egger M S.J. P., Gøtzsche P.C., Vandenbroucke J.P., Initiative S. (2008). The Strengthening the Reporting of Observational Studies in Epidemiology (STROBE)statement: Guidelines for reporting observational studies. J. Clin. Epidemiol..

[37] Tong A., Sainsbury P., Craig J. (2007). Consolidated criteria for reporting qualitative research (COREQ): A 32-item checklist for interviews and focus groups. Int. J. Qual. Healthc.: J. Int. Soc. Qual. Healthc..

